# Role of urinary biomarkers in the diagnosis of congenital upper urinary tract obstruction

**DOI:** 10.4103/0970-1591.42611

**Published:** 2008

**Authors:** Ahmed A. Shokeir

**Affiliations:** Urology and Nephrology Center, Mansoura, Egypt

**Keywords:** Children, kidney, obstruction, urine

## Abstract

**Background:**

Congenital obstructive uropathy constitutes a significant cause of morbidity in children. Currently, there is no reference standard for the diagnosis of renal obstruction in children. The noninvasive measurement of biomarkers in voided urine has considerable appeal as a potential application in children with congenital obstructive nephropathy. The aim of the present review is to explore the current role of biomarkers in the diagnosis and follow-up of obstructive uropathy in children.

**Materials and Methods:**

The literature database (PubMed) was searched from inception to May 2007 regarding the role of urinary biomarkers in the diagnosis and follow-up of children with congenital obstructive uropathy.

**Results:**

The review included 23 experimental and 33 prospective controlled clinical studies. Several cytokines, peptides, enzymes and microproteins were identified as major contributors to or ensuing from obstruction-induced renal fibrosis and apoptosis. The most important biomarkers were transforming growth factor-β_1_ (TGFβ_1_), epidermal growth factor (EGF), endothelin-1 (ET-1), urinary tubular enzymes [N-acetyl-β-D-glucosaminidase (NAG), γ-glutamyl transferase (GGT) and alkaline phosphatase (ALP)], and microproteins [β_2_-microglobulin (β_2_M), microalbumin (M. Alb) and micrototal protein (M.TP)]. All biomarkers showed different degrees of success but the most promising markers were TGFβ_1_, ET-1 and a panel of tubular enzymes. These biomarkers showed sensitivity of 74.3% to 100%, specificity of 80% to 90% and overall accuracy of 81.5% to 94% in the diagnosis of congenital obstructive uropathy in children. Moreover, some of the markers were valuable in differentiation between dilated non-obstructed kidneys in need of conservative management and obstructed kidneys in need of surgical correction. Some studies demonstrated that urinary biomarkers are helpful in the evaluation of success of treatment of children with congenital renal obstruction. Some limitations of the previous studies include lack of different types of controls and small sample size. Larger studies with variable controls are invited to confirm the clinical usefulness of biomarkers in the diagnosis and follow-up of children with congenital obstructive uropathy.

**Conclusion:**

Urinary biomarkers are a promising tool that could be used as a noninvasive assessment of congenital renal obstruction in children.

## INTRODUCTION

Ureteropelvic junction obstruction (UPJO) is the most common cause of hydronephrosis in children. In the past three decades. We have learned that hydronephrosis is not synonymous with obstruction. The differentiation between a dilated obstructed and dilated non-obstructed kidney is a difficult and perplexing problem. No reference standard is available to identify obstruction, and the diagnosis is usually achieved through repeating the various radiological investigations available, such as diuretic ultrasonography (US), radioisotope renography, and/or excretory urography. Nevertheless, radiological investigations expose the child to radiation and may need injection of radiocontrast or radioisotope materials.

The treatment options for UPJO are limited, and are complicated by the fact that the condition resolves in some children and not in others. Thus, predicting which cases of UPJO will resolve spontaneously and which will require surgery is a problem worth investigating.[1–3]

The noninvasive nature of urinary biomarkers gives them significant appeal in their potential application in the diagnosis of UPJO. The aim of the present review is to investigate the role of the different urinary biomarkers in the diagnosis and follow-up of children with upper urinary tract obstruction.

## MATERIALS AND METHODS

The literature database (PubMed) was searched from inception to May 2007 regarding the role of urinary biomarkers in the diagnosis and follow-up of children with congenital obstructive uropathy.

## RESULTS

The review included 23 experimental and 33 prospective controlled clinical studies. Several cytokines, peptides, enzymes and microproteins were identified as major contributors to or ensuing from obstruction-induced renal fibrosis and apoptosis. The most important biomarkers were transforming growth factor-β_1_ (TGFβ_1_), epidermal growth factor (EGF), endothelin-1 (ET-1), urinary tubular enzymes [N-acetyl-β-D-glucosaminidase (NAG), γ-glutamyl transferase (GGT) and alkaline phosphatase (ALP)], and microproteins [β_2_-microglobulin (β_2_M), microalbumin (M.Alb) and micrototal protein (M.TP)]. Each biomarker will be reviewed regarding its pathophysiologic correlation with urinary obstruction and its role in the diagnosis and follow-up of upper urinary tract obstruction as shown in both experimental as well as clinical studies.

### *Transforming growth factor-*β*1 (TGF-*β_*1*_)

#### Pathophysiologic background

TGF-β_1_ is the main modulator of the healing process after tissue injury. Normally, its release ceases by feedback mechanisms when the healing process has been completed, but if TGF-β_1_ release is not switched off, extracellular matrix components are accumulated and tissue fibrosis occurs.[4] Up-regulation of TGF-β_1_ synthesis in the kidney is followed by accumulation of collagen and scarring.[5] It could participate as a key factor in the common mechanisms leading to tissue fibrosis and the development of advanced chronic renal disease of various causes,[6] whereas the administration of specific antiserum against TGF-β_1_ results in amelioration of renal damage.[7]

The response of the upper urinary tract to obstruction involves the induction of a cascade of molecular events and histological changes which involve the up-regulation of the rennin-angiotensin system, with a resultant increase in the expression of tissue TGF-β_1_.[8,9] Honkanen *et al.*[10] proposed that persistently high TGF-β_1_ excretion correlated with morphological indices of chronicity, and the highly increased excretion suggested a persistently active and/or progressive clinical course, whereas lower values suggested a normal situation and remission. This presumes that urinary TGF-β_1_ reflects ongoing sclerotic and fibrotic processes in the kidneys, and that its level could be used as a noninvasive tool to assess the progression of renal disease and to follow the effects of treatments.

#### Experimental studies

Walton *et al.*[11] showed that TGF-B_1_ expression in the obstructed kidneys gradually increased with time following unilateral ureteral obstruction (UUO) of adult Sprague-Dawley rats. Moreover, Chuang *et al.*[9] demonstrated a linear increase of renal gene expression of TGF-β_1_ during the first month of life following UUO in the neonatal rat. In addition, in fetal sheep with hydronephrosis, levels of TGF-β_1_-mRNA were found higher in hydronephrotic kidneys as compared with normal kidneys.[12] Finally, Seseke *et al.*[13] showed that TGF-β_1_ expression was markedly higher in rats with hydronephrotic kidneys, whereas contralateral kidneys did not differ significantly from control values.

#### Clinical studies

At least three clinical studies demonstrated that the mean urinary TGF-β_1_ levels from the dilated renal pelvis were greater than the mean levels in bladder urine of children with UPJO.[14–16]

In a recent clinical study, Taha *et al.*[16] showed that the threshold value of 190 pg/mg creatinine of TGF-β_1_ in voided urine gave a sensitivity of 100%, a specificity of 80% and an overall accuracy of 90.8% in the diagnosis of UPJO in children. The same study also showed that TGF-β_1_ could be used as a noninvasive tool in the long-term follow-up after pyeloplasty of children with UPJO.

Nevertheless, TGF-β_1_ is not specific for obstructive uropathy. Increased urinary TGF-β_1_ excretion was reported in patients with IgA nephropathy,[17] nephritic patients with membranous nephropathy[10] and in patients with insulin-dependent or- independent diabetes mellitus.[18,19]

### Epidermal growth factor

#### Pathophysiologic background

Epidermal growth factor (EGF) is one of the well-known polypeptide growth factors which plays a fundamental role in the regulation of cell proliferation and differentiation.[20] Epidermal growth factor is a mitogen for a variety of renal cells and has important functional effects on intact glomeruli, proximal tubules and collecting ducts. The EGF is a powerful trophic factor for tubular epithelial cells;[21,22] it is normally synthesized by distal tubular cells, with increasing expression during maturation.[23]

The maturation and proliferation of kidney cells occurs through the potential role of the EGF receptor (EGFR) and its ligand in cell division. Lin *et al.*[24] stated that EGFR and its ligand might function together as a transactivation complex, and this can bind to specific DNA sequences to activate the gene expression required for highly proliferative activities. Thus, reductions in EGF levels might reflect reduced EGFR signaling.

#### Experimental studies

Chronic UUO suppresses renal EGF production in neonatal rats.[23] Exogenous EGF reduces tubular apoptosis by 80% in the neonatal rat with chronic UUO, and enhances recovery after the relief of obstruction. Exogenous EGF also inhibits tubular apoptosis in the adult rat subjected to UUO, while in neonatal wild-type mice, exogenous EGF promotes apoptosis instead of cell survival in the obstructed kidney.[25]

#### Clinical studies

It has been demonstrated that children with UPJO have marked reduction in EGF gene expression in the harvested renal tissues during surgery when compared with the expression in controls.[26,27] Grandaliano *et al.*[28] reported significantly less urinary EGF in a group of children with UPJO than in controls; a finding not supported by a recent study by Taha *et al.*[16] With a cut-off value of 40 ng/mg creatinine in voided urine, EGF gave a sensitivity of 40%, a specificity of 80% and an overall accuracy of 58.5% in the diagnosis of UPJO in children.[16] Therefore, EGF is currently considered of low clinical importance in the diagnosis of upper urinary tract obstruction.[16]

### Endothelin - 1 (ET-1)

#### Pathophysiologic background

ET-1 is the most potent and powerful endogenous vasoconstrictive peptide known today and it is 10 times more potent than angiotensin II.[29] ET-1 has been implicated in the tissue damage and dysfunction associated with UUO.[30] Kelleher *et al.*[31] demonstrated that ET-1 has a predominant role in the development of preglomerular arteriolar stenosis in the obstructed upper urinary tract. Additionally, results have suggested that ET-1 may play a role in the progression of interstitial fibrosis after ureteral ligation.[32] It has also been shown that ET-1 levels are higher in the renal vein than in the arterial inflow in UUO, suggesting that ET-1 production is renal in origin, rather than systemic.[33] The blockade of the ET-1 receptors prevents renal dysfunction and attenuates the decrease in renal plasma flow and glomerular filtration rate in rats subjected to ureteral obstruction.[34]

#### Experimental studies

Hegarty *et al.*[35] performed a semiquantitative analysis of ET-1 expression in rats subjected to UUO and showed an increase in ET-1 expression in the obstructed kidney with decreased expression in the contralateral kidney compared with the sham-operated control. They also administered bosentan (an ET-1 receptor antagonist) to a group of obstructed animals and observed an inhibition of ET-1 receptors in this group of rats that was associated with restoration of blood flow in the obstructed kidney and a reduction of the apoptotic rate to values similar to that in the control kidney. The magnitude of these restorative effects would implicate ET-1 as a principal mediator of vascular and cellular injures in UUO.[35]

Miller *et al.*[36] studied the gene expression of ET-1 in rats with congenital unilateral UPJO. They found that the gene expression of ET-1 in the renal pelvis and UPJ of kidneys with UPJO was significantly elevated compared with the expression in the renal pelvis and UPJ of the kidneys of healthy rats. They concluded from these observations that the increased ET-1 expression in UPJO may suggest a pathogenic role for this peptide in ureteral obstruction.

#### Clinical studies

Knerr *et al.*[37] studied the gene expression of ET-1 in the stenotic tissue of congenital UPJO in children. They showed that the gene expression of ET-1 in the obstructed UPJ was significantly greater than in the control tissue.

Taha and associates[38] are the first to measure the urinary ET-1 level in children with UPJO. They have shown that the voided urine ET-1 level in children with UPJO is significantly elevated up to fourfold of that in the controls. This means that bladder ET-1 could be used clinically to confirm the diagnosis of UPJO in children. A cutoff value of 3 fmol/mg creatinine gave a sensitivity of 74.3%, a specificity of 90% and an overall accuracy of 81.5%.

### Urinary enzymes

#### Pathophysiologic background

Obstructive nephropathy involves detrimental changes in the proximal tubules of the affected kidney, leading to damage in the cell membranes resulting in release of lysosomal enzymes like N-acetyl-β -D-glucosominidase (NAG) and brush border enzymes like γ-glutamyl transferase (GGT) and alkaline phosphatase (ALP). The appearance of these tubular enzymes in urine has been proven to be a valuable marker of damage to the proximal tubules.[39]

NAG is the most widely assayed urinary enzyme for the detection of renal damage and the diagnosis of renal disease. This is due to its stability in urine, its relatively large molecular mass (130 KDa) which precludes filtration by the glomerulus and its presence in high activity in the tubular lysosomes.[40] Therefore, elevation of NAG activity in urine provides a marker for renal tubular damage or more precisely loss of lysosomal integrity.[41] Because of the location of GGT in the brush border of the proximal tubule, the urinary assay of this enzyme in cases of obstructive nephropathy has proved to be reliable as a marker of the luminal membrane function of such a segment.[42]

#### Experimental studies

Urinary NAG activities in urine of Wister rat kidneys subjected to a stable partial ureteral obstruction were found to be significantly higher as compared with that of the contralateral control kidneys during the first two weeks of partial ureteral obstruction.[43,44] However, urinary GGT activities did not show such clear-cut differences between hydronephrotic obstructed kidneys and contralateral control kidneys.[43]

#### Clinical studies

Several clinical studies demonstrated that the activities of the three tubular enzymes (NAG, GGT and ALP) in urine collected from the dilated renal pelvis during surgery in children with UPJO were consistently higher than those seen in their bladder urine.[45,46]

A recent article in the Hungarian literature revealed that the activities of NAG, ALP and GGT in the urine of children with upper obstructive uropathy were two to 10 times higher compared to these in normal children.[47] Taha *et al.* have also shown a significant increase in the activities of NAG, ALP and GGT in the voided urine of children with UPJO, with levels up to 2.34-fold those in children with dilated non-obstructed kidneys.[46] This finding means that voided urinary enzymes could be used clinically to support the diagnosis of UPJO in children.

A recent study determined the cutoff values of urinary NAG, ALP and GGT giving the highest diagnostic field in the setting of UPJO in children.[46] A cutoff value of 7.8 mu/mg creatinine NAG yielded a sensitivity of 97.1%, a specificity of 80% and an overall accuracy of 92%. A cutoff value of 34.5 IU/gm creatinine ALP resulted in a sensitivity of 91.4%, a specificity of 100% and an overall accuracy of 94%. A cutoff value of 54 IU/gm creatinine GGT yielded a sensitivity of 62.9%, a specificity of 100% and an overall accuracy of 74%. The combination of NAG and ALP resulted in a sensitivity of 100%, a specificity of 80% and an overall accuracy of 94%. Notably, despite having the same origin, ALP and GGT demonstrated different sensitivities, possibly because ALP enzyme is localized more superficially in the brush border compared to GGT, which is localized more deeply in the membrane.[48]

These tubular renal enzymes provide a high level of sensitivity but only a moderate level of specificity. It is noteworthy that urinary NAG levels were consistently increased in other clinical conditions, such as high-grade reflux, urinary tract infection, glomerulonephritis and diabetes mellitus.[49]

The observation that NAG, ALP and GGT in the urine of children with UPJO are markedly increased in comparison to dilated non-obstructed controls is important in the differentiation between dilated obstructed and dilated non-obstructed pelvi-calyceal systems in children with congenital hydronephrosis. This differentiation will help the urologist to choose between conservative management and surgical intervention, although there are no specific standards to unequivocally indicate surgery. Further prospective comparative studies in larger patient populations are needed to justify the role of these urinary enzymes in the diagnosis of UPJO in children.

Tataranni *et al.*[42] followed the recovery of tubules after relief of obstructive nephropathy in adults and found that the urinary NAG output remained increased for as long as 45 days after resumption of diuresis. Taha *et al.*[46] also observed that a duration of three to six months is required for the three biomarkers to show significant reduction in their activities in comparison to preoperative basal activities in children with UPJO. This finding indicates that the kidney takes time to achieve functional and ultrastructural recovery after relief of obstruction.

A recent study demonstrated a perfect negative correlation between the function of the corresponding kidney and urinary biomarkers indicating that the measurement of these enzymes in voided urine could be used as a noninvasive tool for long-term follow-up of children with UPJO after pyeloplasty and those receiving conservative treatment.[46] On the other hand, Carr *et al.*[45] demonstrated that the severity of obstruction as determined radiographically did not always agree with the NAG activity, but that the modality agreeing best with the biochemical findings was renal ultrasonography.

### Microproteins

#### Pathophysiologic background

One of the main functions of the glomerulus is the selective filtration of plasma proteins. Low molecular weight proteins e.g. β_2_-microglobulin are completely filtered by the glomerulus and reabsorbed by the tubules. Therefore, the presence of β_2_-microglobulin in urine is considered as a sign of tubular dysfunction. On the other hand, high molecular weight proteins (>40 KDa) e.g. microalbumin and micrototal protein are not filtered by the glomerulus in normal conditions. Glomerular permeability increases as a consequence of inflammation or basement membrane damage due to obstructive uropathy and there will be an increase in the filtration of high molecular weight proteins. Therefore, the presence of microalbumin and micrototal proteins in urine is considered a sign of glomerular dysfunction.[50–52]

#### Experimental studies

Measurement of β_2_-microglobulin in urine was used for the assessment of tubular dysfunction. In the experimental studies, the urinary β_2_-microglobulin/urinary creatinine ratio was found to be significantly elevated one week after the occurrence of unilateral total ureteral obstruction in Wister rats as compared to controls.[39] Moreover, significant increases in urinary β_2_-microglobulin levels were also found in rats subjected to unilateral and bilateral partial ureteral obstruction.[53]

Urinary excretion of high molecular weight proteins is a marker of glomerular dysunction and glomerular proteinuria is considered the most common and serious type of proteinuria.[51] Urinary microalbumin/creatinine ratio was found to be significantly elevated one week after the occurrence of UUO in Wister rats as compared to controls.[39]

#### Clinical studies

The level of β_2_-microglobulin in urine collected from dilated renal pelvis during surgery in children with UPJO was found to be consistently higher than that seen in their bladder urine.[45] Moreover, the urinary β_2_-microglobulin level was found to be significantly elevated in patients with UPJO as compared to controls. This elevation remained for three months after relief of obstruction and showed marked and rapid decrease between three to four months post surgery.[42]

Lama *et al.*[54] demonstrated that the urinary levels of microalbumin and micrototal proteins were significantly higher in the voided urine obtained from children with UPJO as compared to the levels in non-obstructed controls. The level of microalbumin continued to increase during follow-up after surgery and its value started to decrease after 18 months following pyeloplasty.[54]

## DISCUSSION

Congenital UPJO constitutes a significant cause of morbidity in children and exists in a wide range of severity and clinical manifestations. It produces a variety of renal parenchymal changes which may, in part, reflect abnormal development. When untreated, it will impair nephron growth and function causing progressive renal deterioration.[54] Currently, there is no gold standard for the assessment of renal obstruction to which we can compare an individual case. The diagnosis in most cases is only possible by repeated investigations and comparing changes of the parameters during a larger follow-up. Of these investigations are grey-scale US, Doppler US, radioisotope renography, excretory urography, contrast-enhanced computed tomography and magnetic resonance urography. Each of these modalities has its own merits and disadvantages, but none of them is ideal.[55]

A biochemical marker in the urine that could provide information to the obstructive nature of hydronephrosis would reduce the degree of invasiveness, subjectivity and operator-dependent proficiency required of the currently available radiological modalities.[14] So, the clinical usefulness of a bladder urine biomarker for aiding in the diagnosis of upper urinary tract obstruction is obviously appealing.

There is considerable structure and function specialization between the different regions of the nephron which are characterized by the presence of 13 different cell types.[56] As a result of the specialization of the different regions of the kidney, damage to a specific region would result in characteristic changes in the profile of biomarkers in the urine. Progression to more widespread damage would tend to result in a uniform profile of urinary biomarkers reflecting damage to different regions.[57]

In the present review, all biomarkers showed different degrees of success but the most promising markers were TGFβ_1_, ET-1 and a panel of tubular enzymes. These biomarkers showed sensitivity of 74.3% to 100%, specificity of 80% to 90% and overall accuracy of 81.5% to 94% in the diagnosis of congenital obstructive uropathy in children. Moreover, some of the markers were valuable in differentiation between dilated non-obstructed kidneys in need of conservative management and obstructed kidneys in need of surgical correction. In addition, some studies demonstrated that urinary biomarkers are helpful in the evaluation of success of treatment of children with congenital renal obstruction.

Nevertheless, the currently available urinary biomarkers are not specific for obstructive uropathy. Increased urinary biomarkers were reported in other diseases such as IgA nephropathy, nephritic patients with membranous nephropathy, high-grade reflux, urinary tract infection, glomerulonephritis and in patients with diabetes mellitus.[18,19]

Notably, some limitations of the already existing literature include lack of different types of controls and small sample size. Larger studies with variable controls are invited to exactly determine the role of biomarkers in the diagnosis and follow-up of children with congenital obstructive uropathy.

## CONCLUSIONS

Urinary biomarkers are a promising tool that could be used as a noninvasive assessment of congenital renal obstruction in children. The most promising markers are TGFβ_1_, ET-1 and a panel of tubular enzymes. These biomarkers are useful not only in the diagnosis of congenital obstructive uropathy but also in the differentiation between dilated non-obstructed kidneys in need of conservative management and obstructed kidneys in need of surgical correction. Moreover, some studies demonstrated that urinary biomarkers are helpful in the evaluation of success of treatment of children with congenital renal obstruction. Nevertheless, the existing literature could be criticized for having small sample size and lacking different types of controls. Larger studies with variable controls are invited to confirm the clinical usefulness of urinary biomarkers in the diagnosis and follow-up of children with congenital obstructive uropathy.
